# Water-Soluble Polymer Assists *N*-Methyl-D-Aspartic Acid Receptor 2B siRNA Delivery to Relieve Chronic Inflammatory Pain In Vitro and In Vivo

**DOI:** 10.1155/2018/7436060

**Published:** 2018-01-14

**Authors:** Jie Peng, Jiahui Ma, Xue Yang, Huan He, Haopeng Wu, Tongtong Ma, Jianhua Lu

**Affiliations:** ^1^Department of Anesthesiology, Guangzhou General Hospital of Guangzhou Military Command of Chinese PLA, Guangzhou 510010, Guangdong, China; ^2^Department of Anesthesiology, Second Affiliated Hospital, Guangzhou University of TCM, Guangzhou 510120, Guangdong, China

## Abstract

We constructed a water-soluble lipopolymer (WSLP) as a nonviral gene carrier to deliver siRNA targeting NR2B. The cytotoxicity and serum stability of WSLP loaded with siRNA were evaluated, and the knockdown efficiency of WSLP/NR2B-siRNA in PC12 cells was examined. The results showed that WSLP could protect the loading siRNAs from enzymatic degradation in serum and exhibit low cytotoxicity to cells. After transfection, WSLP/NR2B-siRNA complexes reduced the NR2B transcriptional level by 50% and protein level by 55% compared to control siRNA. Moreover, 3 days after intrathecal injection of WSLP/NR2B-siRNA complexes into rats, the NR2B protein expression decreased significantly to 58%, compared to control treatment (*p* < 0.01). Injection of WSLP with scrambled siRNA or of polyethylenimine (PEI) with NR2B-siRNA did not show this inhibitory effect. Additionally, injection of WSLP/NR2B-siRNA complexes significantly relieved inflammatory pain in rats at 3, 4, and 5 days with reduced MWT and decreased TWL scores, while injection of WSLP with scrambled siRNA or of PEI with NR2B-siRNA did not. These results demonstrated that WSLP can efficiently deliver siRNA targeting NR2B to PC12 cells and relieve pain in rats with chronic inflammatory pain.

## 1. Introduction

The phrase “neuropathic pain” appeared in public view only in the last decade and has been increasingly appreciated as a frequent source of chronic pain. Neuropathic pain is defined as “pain caused by a lesion or disease of the somatosensory system.” Nerve injury, nerve compression, diabetes, infection, and autoimmune disease may result in neuropathic pain [1].

Overexpression of *N*-methyl-D-aspartic acid receptor 2B (NR2B) plays an important role in the development of hyperalgesia [2]. Pain treatment research has focused on using siRNA targeting NR2B to suppress the expression of NR as an effective way to treat chronic pain [3]. Finding an appropriate transduction tool is critical for the success of siRNA delivery into the human body. To avoid potential safety issues, nonvirus carriers are typically used for in vivo genetic treatment. However, commonly used means of nonvirus carrier treatment, such as liposome-mediated transfection and electroporation, have a desirable effect in vitro but a reduced effect in vivo [4].

RNA interference (RNAi) offers great potential not only for in vitro target validation but also as a novel therapeutic strategy based on the highly specific and efficient silencing of a target gene. Since it relies on small interfering RNAs (siRNAs), which are the mediators of RNAi-induced specific mRNA degradation, a major issue is the delivery of therapeutically active siRNAs into the target tissue/target cells in vivo. For safety reasons, strategies based on (viral) vector delivery may be of only limited clinical use. The more desirable approach is to directly apply catalytically active siRNAs. Viral vectors are efficient gene transduction tools for in vitro cells. In vivo, nonviral vectors delivering siRNA have been used previously [5, 6]. Polyethylenimine (PEI) is an organic material that can be used as a nonviral vector. Unfortunately, high-molecular-weight PEI is cytotoxic. Low molecular weight PEI has low cytotoxicity but also has a low transfection efficiency [7–9]. Recently, cholesterol has been used to modify low molecular weight PEI, and a water-soluble lipopolymer (WSLP) comprising cholesterol and low molecular weight PEI exhibited good biocompatibility, high transfection efficiency, and minimal toxicity [10–13]. A previous study indicated that WSLP can permeate the blood-brain barrier [14]. Therefore, the present study hypothesizes that WSLP delivering siRNA targeting NR2B may inhibit NR2B expression in the spinal dorsal horn and may be used as a novel method for treating neuropathic pain. This WSLP exhibited high transfection efficiency and negligible toxicity, which owned the potential to target the central nervous system (CNS) in vivo [15–18]. Therefore, we hypothesized that WSLP could be a reliable carrier for delivering NR2B-siRNA, which would suppress NR2B expression and consequently relieve chronic pain. Given that NR2B was the main cause of chronic inflammatory pain, in the present study, we attempted to treat the pain by knocking down the expression of NR2B using the WSLP delivery system described above. We prepared a siRNA targeting NR2B and fused it to WSLP, defined as WSLP/NR2B-siRNA. Our data showed that WSLP/NR2B-siRNA efficiently delivered siRNA into PC12 cells. In addition, we evaluated the MWT and TWD scores of the rat model with inflammatory pain treated with intrathecal injection of WSLP/NR2B-siRNA complexes in vivo.

## 2. Materials and Methods

### 2.1. WSLP Synthesis

WSLP was synthesized according to previously described methods [19]. Briefly, 30 mL of dehydrated chloroform (Guangzhou Chemical Reagent Factory, Guangzhou, China) was added to 100 *μ*L of dehydrated trimethylamine and then mixed with 3 g of PEI (molecular weight ∼18,000, Suzhou Jingchun, Suzhou, China). This mixture was placed on ice, stirred for 30 min, mixed with 1 g of cholesterol chloroformate (Sigma-Aldrich Co. LLC, USA), dissolved in 10 mL of chloroform at 0°C, and stirred overnight. A yellow, viscous gel was obtained from this process, and it was dissolved in 50 mL of hydrochloric acid (0.1 mol/L). This dissolved gel was mixed with 200 mL of dehydrated chloroform to remove other polymers and cholesterol, and then, it was filtered through filter membranes (membrane pore size of 30 *μ*m). The abovementioned procedures were repeated twice. The products were freeze-dried under low pressure to yield a yellow powder.

### 2.2. Synthesis of WSLP/siRNA Complexes

Double-stranded NR2B-siRNA was designed and synthesized (Guangzhou RiboBio Co., Ltd., Guangzhou, China). The sequences used were as follows: positive-sense strand of NR2B-siRNA, 5′-GGA UGA GUC CUC CAU GUC UdT dT-3′; antisense strand of NR2B-siRNA, 5′-GAA CAU GGA GGA CUC AUC CdT dT-3′; positive-sense strand of scrambled control siRNA (scRNA), 5′-AGU GGA GUC CUC CAU GUC UdT dT-3′; and antisense strand of scRNA, 5′-AGA CAU GGA GGA CUC CAC UdT dT-3′. WSLP dissolved in diethyl pyrocarbonate- (DEPC-) treated glucose (5%) was mixed with siRNA at mass ratios ranging from 1 : 1 to 7 : 1 for in vitro experiments. The resulting mixtures were incubated for 30 min at room temperature and then separated by electrophoresis in an 1.5% agarose gel at 120 V in TAE buffer for 20 min. The enwrapped siRNA by WSLP was analyzed by a Gel Doc 2000 gel image analysis system (Bio-Rad, Hercules, CA, USA). For in vivo experiments, WSLP was mixed with siRNA at a mass ratio of 5 : 1, based on the results of the in vitro study.

### 2.3. Stability of WSLP/NR2B-siRNA in Serum

WSLP and NR2B-siRNA were mixed at a mass ratio of 5 : 1; equal amounts of the complexes were randomly placed into five centrifugation tubes, and the samples were incubated at room temperature for 30 min. The samples were then infused with 10% aborted calf blood serum and incubated at 37°C for 0, 0.5, 1, 2, and 3 h. Each sample was subsequently electrophoresed as described above for 20 min, and the results were observed using a Gel Doc 2000 gel image analysis system (Bio-Rad, Hercules, CA, USA).

### 2.4. Cytotoxicity Assay

PC12 cells were seeded at a density of 2000 cells/well in a 96-well plate and transfected with WSLP/NR2B-siRNA complexes at mass ratios of 1 : 1, 2 : 1, 3 : 1, 5 : 1, 10 : 1, or 20 : 1, respectively. Next, 20 *μ*L of 3-(4,5-dimethylthiazol-2-yl)-5-(3-carboxymethoxyphenyl)-2-(4-sulfophenyl)-2H-tetrazolium (MTS) was added to each well, and the samples were incubated at room temperature for 4 h. The optical density (OD) was measured at 570 nm using a microplate reader (Biocell, USA). Higher OD values indicated higher levels of cellular viability. Nontransfected PC12 cells were used as a negative control.

### 2.5. Transfection of WSLP/NR2B-siRNA to Silence the NR2B

PC12 cells were randomly divided into three groups: the negative transfection group (NT group), transfected with NR2B-siRNA; the control transfection group (CT group), transfected with WSLP/scRNA; and the WSLP transfection group (WT group), transfected with WSLP/NR2B-siRNA. PCR and western blot assays were used to detect the mRNA and protein expression levels of NR2B in cells.

### 2.6. Animals

A total of 100 healthy male Sprague-Dawley rats aged 6 weeks and weighing 180–200 g were provided by the Animal Experimental Center of the Guangzhou General Hospital of the Guangzhou Military Command of the Chinese People's Liberation Army (no. SYXK(Yue)2009-0100). Rats of both sexes were bred and maintained in a specific pathogen-free unit at room temperature with humidity regulated (21 ± 2°C; 55 ± 10%) under 12/12 h light/dark cycle with lights off at 19:30 h and no twilight period. Mice were housed in individually ventilated cages (IVCs) (Tecniplast Sealsafe 1284L) receiving 60 air changes per hour, at a stocking density of 4-5 rats per cage unless otherwise stated below (overall dimensions of caging (*L* × *W* × *H*): 398 × 215 × 187 mm, floor area = 530 cm^2^). Aspen bedding substrate and standard environmental enrichment of nestlet, cardboard tunnel, and three wooden chew blocks were provided. Rats were given water and breeding diet (irradiated A03, SAFE, France) ad libitum unless otherwise stated.

About 100 rats were randomly assigned to five groups (20 rats per group): the control group (NS group), in which the rats received no treatment; the chronic inflammatory pain model group (CFA group), in which the rats had inflammatory pain induced by intraplantar injection of complete Freund's adjuvant (CFA); the PEI/NR2B-siRNA group (PEI group), in which the rats were subjected to the same chronic inflammatory pain model but also received an intrathecal injection of low molecular weight PEI/NR2B-siRNA; the WSLP/NR2B-siRNA group (WSLP group), in which the rats were subjected to the same chronic inflammatory pain model but also received an intrathecal injection of WSLP/NR2B-siRNA; and the WSLP/scRNA group (sWSLP group), in which the rats were subjected to the same chronic inflammatory pain model but also received an intrathecal injection of WSLP/scRNA.

This study received permission from the Animal Ethics Committee of the Guangzhou General Hospital of the Guangzhou Military Command of the Chinese PLA, China.

### 2.7. Model Establishment and Intrathecal Injection of WSLP/siRNA

The inflammatory pain model was established by injecting 120 *µ*L of CFA (1 g/L) into the footpad of the rats. The NS group was administered an intraplantar injection of 120 *μ*L of 0.9% normal saline instead of CFA. Chronic inflammatory pain rat models were done by subcutaneous injection of complete Freund's adjuvant (CFA) at the right rear toe. Briefly, the rats were anesthetized using 4% chloral hydrate (1 ml/100 g i.p.) and then subcutaneous injection of 120 *μ*L CFA in the pain model rats and PBS in control models. The exclusion criteria were used to remove failed model rats as follows: the values of TWL and MWT were measured at day 1 after modeling, and the values decreased significantly proved successful modeling and these rats were included; vice verse, they were excluded from the experiments.

Various drugs were intrathecally administered according to previously described methods [20]. The drugs were slowly injected using a microsyringe vertically aligned to the L5-6 space. Successful intrathecal punctures were demonstrated by rats exhibiting a tail tremble or sudden lateral swing. One day after model establishment, the rats in the PEI, WSLP, and sWSLP groups received a single intrathecal injection of 20 *μ*l of PEI/siRNA, WSLP/siRNA, or WSLP/scRNA, respectively.

The mechanical withdrawal threshold (MWT) and thermal withdrawal latency (TWL) of 10 rats from each group were measured in the morning 1 day before model establishment as well as 3, 4, and 5 days after intrathecal injection. The other 10 rats from each group were intraperitoneally injected with 4% chloral hydrate 3 days after the intrathecal injection, and their spinal dorsal horns at L4 were subsequently harvested on ice and divided equally for PCR and western blot assays.

### 2.8. qPCR

The PCR primers used to detect NR2B were as follows: NR2B upstream, 5′-TGC ACA ATT ACT CCT CGA CG-3′, and NR2B downstream, 5′-TCC GAT TCT TCT TCT GAG CC-3′. The resulting NR2B PCR products were 222 bp long. *β*-actin was used as an internal reference, and its upstream and downstream primers were 5′-TCA TGA AGT GTG ACG TTG ACA TCC GTA AAG-3′ and 5′-CCT AGA ATT TGC GGT GCA CGA TGG AGG-3′, respectively. The *β*-actin PCR products were 409 bp long.

All primers were synthesized by Shanghai Sangon Biotech, Shanghai, China. RNA was extracted and mixed with 0.5 *μ*L RNase inhibitor, 2.0 *μ*L dNTP, 2.0 *μ*L oligo (dT) 15, 4.0 *μ*L 5× buffer, 0.5 *μ*L DEPC-DW, and 1.0 *μ*L reverse transcriptase at 37°C for 1 hour and then at 70°C for 10 minutes to synthesize cDNA. Four-microliter cDNA, 5 *μ*L 10× buffer, 1 *μ*L dNTP, 1 *μ*L primers, 1 *μ*L *β*-actin, 32.5 *μ*L ddH2O, 3 *μ*L MgCl_2_, and 0.5 *μ*L Taq enzyme were mixed and reacted at 94°C for 30 seconds, 55°C for 30 seconds, and 72°C for 30 seconds for a total of 30 cycles, followed by extension at 72°C for 5 minutes. The PCR products were electrophoresed in an 1.5% agarose gel, and the electrophoresed gel was analyzed using a Gel Doc 2000 gel image analysis system (Bio-Rad, Hercules, CA, USA). The ratio of NR2B absorbance to *β*-actin absorbance was used to quantify the expression.

### 2.9. Western Blot

Seventy-two hours after transfection, the incubation media were discarded, and the cells were washed 2-3 times with precooled PBS. The cells were then placed in microcentrifugation tubes, 400 *μ*L of radio immunoprecipitation assay (RIPA) lysate and 4 *μ*L of phenylmethylsulfonyl fluoride (PMSF) were added to each tube on ice, and the cells were lysed on dry ice for 30 min. Finally, the cells were centrifuged at 12,000 ×g for 30 min at 4°C, and the resulting supernatants were stored at −80°C.

Spinal dorsal horns at L4–6 were ground into precooled tissue lysates at 4°C, placed in an ice bath for 5 minutes, and centrifuged at 800 ×g for 15 min. The supernatants were harvested, and their protein concentrations were measured using the bicinchoninic acid assay method as previously described [21]. Sodium dodecyl sulfate polyacrylamide gel electrophoresis (with a separation gel concentration of 4% and a stacking gel concentration of 6%) was performed with 80 *μ*g of samples in each lane. The proteins were semidried, transferred to polyvinylidene difluoride membranes, and blocked with 5% nonfat milk powder. The membranes were incubated with a goat anti-rat NR2B monoclonal antibody (Santa Cruz Biotechnology, Santa Cruz, CA, USA) in hybridization solution (Santa Cruz Biotechnology, Santa Cruz, CA, USA) (1 : 500) for 2 h, followed by incubation with horseradish peroxidase-labeled rabbit anti-goat IgG hybridization solution (1 : 5000) for 1 h. The products were subjected to enhanced chemiluminescence and imaging with ultraviolet light. The target protein bands were analyzed using a Gel Doc 2000 gel image analysis system (Bio-Rad). The product of absorbance and area (OD × mm^2^) was used to quantify protein expression.

### 2.10. Detection of Pain Behaviors

MWT was determined using a Von Frey stimulation device (Von Frey Kit Cat. no. 1277, Ugo Basile Biological Research Apparatus, Varese, Italy). MWT detection was performed between 09:00 and 12:00 in a quiet environment. 30 min prior to MWT detection, each rat was placed in a transparent organic glass container to adapt to the environment. Von Frey cilia of different sizes were used to vertically stimulate the sole of the hind foot for 6–8 s. Escape reactions included elevating the hind foot, delaying in putting down the hind foot, trembling, licking the hind foot, or escaping. The 50% withdrawal threshold was calculated [22].

TWL was determined using a plantar test (Plantar Test Cat. no. 37,370, Ugo Basile Biological Research Apparatus, Varese, Italy). The stimulus intensity was controlled at a range where the withdrawal reaction occurred at 12–15 s following the stimulation. The thermal stimulation lasted for 30 s. The interval between stimulations was 5 min. The detection was repeated in triplicate, and the mean value was calculated.

### 2.11. Statistical Analysis

All statistical analyses were performed with Statistical Package for the Social Sciences (SPSS) 13.0 software. The data are presented as the mean ± SD from three separate experiments. Statistical significance was determined by the paired or unpaired Student's *t*-test in cases of standardized expression data. Differences were considered statistically significant at *p* < 0.05. We have added this paragraph in the Method section.

## 3. Results

### 3.1. Feasibility and Stability of Polyelectrolyte Complex Formed by WSLP with siRNA

**Figure 1:**
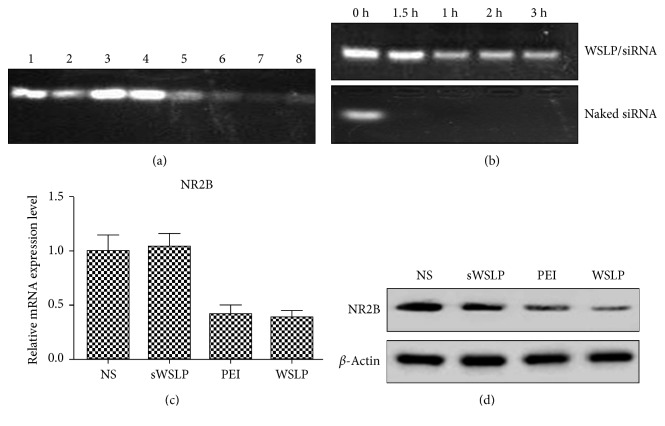
Complex formation of WSLP/siRNA. (a) Determination of siRNA in WSPL/siRNA complexes in different weight ratios by using 20% PAGE gel. Lanes 1–8: naked siRNA, 1 : 1, 2 : 1, 3 : 1, 4 : 1, 5 : 1, 6 : 1, and 7 : 1, respectively. (b) The stability of WSPL/siRNA complex of 2 : 1 in serum. (c) NR2B mRNA expression was tested by qRT-PCR as normalized to *β*-actin. (d) NR2B protein expression was analyzed by WB.

To optimize the ratio between WSLP and siRNA in a complex, we determined the enwrapped siRNA by using electrophoresis. WSLP was conjugated with NR2B-siRNA at different mass ratios ranging from 1 : 1 to 7 : 1 and subjected the samples to electrophoresis. As shown in Figure 1(a), the brightness of the siRNA bands gradually faded with the increase of WSLP/siRNA ratios from 1 : 1 to 7 : 1. At a ratio of 5 : 1, the siRNA band was as weak as most of the siRNA in 6 : 1 and 7 : 1. Hence, we prepared the WSLP/siRNA complex at a ratio of 5 : 1 for future analysis (Figure 1(a)).

Then, the stability of the complex in serum was evaluated, as it was previously reported that WSLP conjugation would protect nucleic acid from degradation. Based on the serum stability of PEI-siRNA, control was previously confirmed several times by other researchers [23], and we did not repeat the examination. The naked siRNA was degraded in serum within 1.5 h, while the WSPL-conjugated siRNA could stay stable as long as 3 h in serum (Figure 1(b)). These results suggested that the WSLP/siRNA could be used as a stable siRNA delivery tool.

In addition, the mRNA and protein levels of NR2B were further detected by qRT-PCR and WB, respectively. The results showed that there were no obvious alterations between the NS and sWSLP groups in NR2B expressions, while NR2B expressions in mRNA and protein levels in the PEI and WSLP groups were notably decreased as compared to the NS and sWSLP groups (Figures 1(c) and 1(d)).

### 3.2. Cytotoxicity of WSLP/siRNA In Vitro

**Figure 2:**
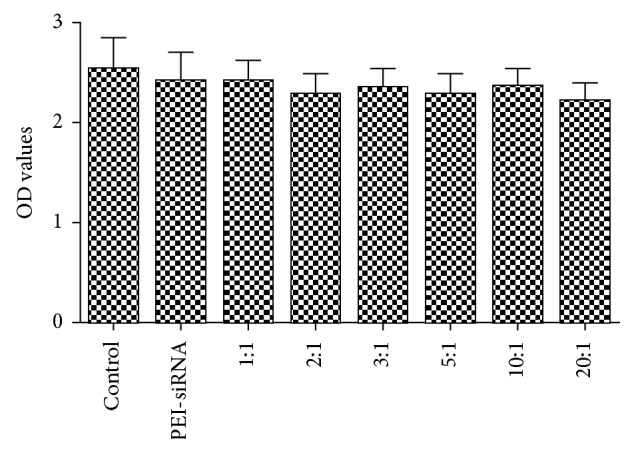
Assessment WSLP/NR2B-siRNA cytotoxicity. MTS assays were performed on untransfected PC12 cells and PC12 cells transfected with PEI-siRNA and varying proportions of WSLP/NR2B-siRNA (1 : 1, 2 : 1, 3 : 1, 5 : 1, 10 : 1, or 20 : 1). Error bars represent the standard deviation. Each experiment was repeated 3 times.

As a useful siRNA delivery tool, low cytotoxicity to cells is equally important for high efficiency. Next, we aimed to assess the cytotoxicity of WSLP/siRNA in vitro. We performed an MTS assay on untransfected PC12 cells (control group) and PC12 cells transfected with PEI-siRNA and various proportions of WSLP/NR2B-siRNA (1 : 1, 2 : 1, 3 : 1, 5 : 1, 10 : 1, and 20 : 1). We found that the resulting ODs of the PC12 cells transfected with any of the tested proportions of WSLP/NR2B-siRNA were not significantly different (*p* > 0.05) from those of PC12 cells transfected with PEI-siRNA and untransfected PC12 cells (Figure 2), confirming that WSLP/NR2B-siRNA complexes are not cytotoxic in these proportions.

### 3.3. NR2B Silencing by WSLP/siRNA Complexes In Vitro

To confirm that NR2B-siRNA is able to effectively silence NR2B when it is complexed with WSLP, we transfected PC12 cells with NR2B-siRNA (NT group), WSLP/scRNA (CT group), or WSLP/NR2B-siRNA (WT group) and assessed their NR2B mRNA and protein levels with PCR and western blot assays, respectively. The PCR results showed that the NR2B mRNA level of the WT group was significantly lower than that of the NT group, with a silencing rate of 50% (*p* < 0.01). The western blot results similarly showed that the average NR2B protein expression level of the WT group was 55% lower than that of the NT group (*p* < 0.01). There was no significant difference in the NR2B mRNA level or protein expression level between the NT and CT groups (*p* > 0.05; Table 1). These results show that WSLP/NR2B-siRNA silences NR2B more effectively than NR2B-siRNA that is not complexed with WSLP.

### 3.4. NR2B Silencing by WSLP/siRNA Complexes In Vivo

After finding that the WSLP/NR2B-siRNA complexes could knock down NR2B expression in vitro, we tested if they could also silence NR2B expression in vivo by using a rat model of chronic inflammatory pain.

**Figure 3:**
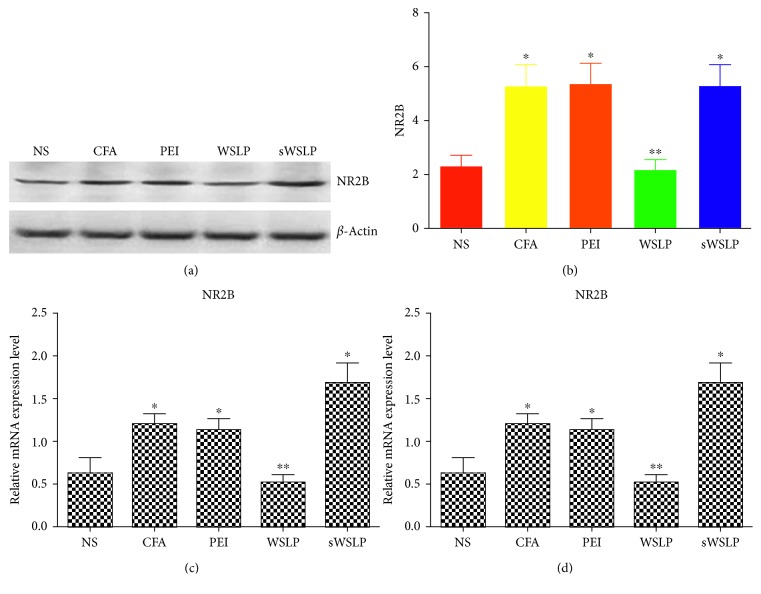
Effects of various intrathecal treatments on NR2B expressions at protein and mRNA levels in the spinal dorsal horn of rats with chronic inflammatory pain. Rats were untreated (NS) or injected with CFA to establish a model of inflammatory pain and subsequently injected with a control treatment (CFA), a combination of PEI and NR2B-siRNA (PEI), WSLP/NR2B-siRNA (WSLP), or WSLP/scRNA (sWSLP). (a) Representative images of the NR2B (top) and *β*-actin (bottom) protein levels were showed by WB detection. (b) Quantification of the NR2B protein expression was displayed with a histogram. Values shown are the mean ± standard deviation of ten rats per group. (c) NR2B mRNA expression was examined by qRT-PCR. ^∗^*p* < 0.05 versus NS group; ^∗∗^*p* < 0.01 versus CFA group.

We found that compared to the control NS group, the CFA, PEI, WSLP, and sWSLP groups showed significantly higher NR2B protein levels 3 days after administration of the intrathecal injection (*p* < 0.01; Figure 3(a)). Additionally, compared to the CFA group, the WSLP group showed 58% lower NR2B protein levels (*p* < 0.01), but the NR2B expression in the PEI and sWSLP groups was similar to that in the control group (*p* > 0.05; Figure 3(b)). In addition, the expression pattern of NR2B mRNA levels at 3 days after administration of the intrathecal injection in different groups was similar to its protein levels (Figure 3(c)).

### 3.5. Intrathecal Injection of WSLP/NR2B-siRNA Relieved Inflammatory Pain in Rats

**Figure 4:**
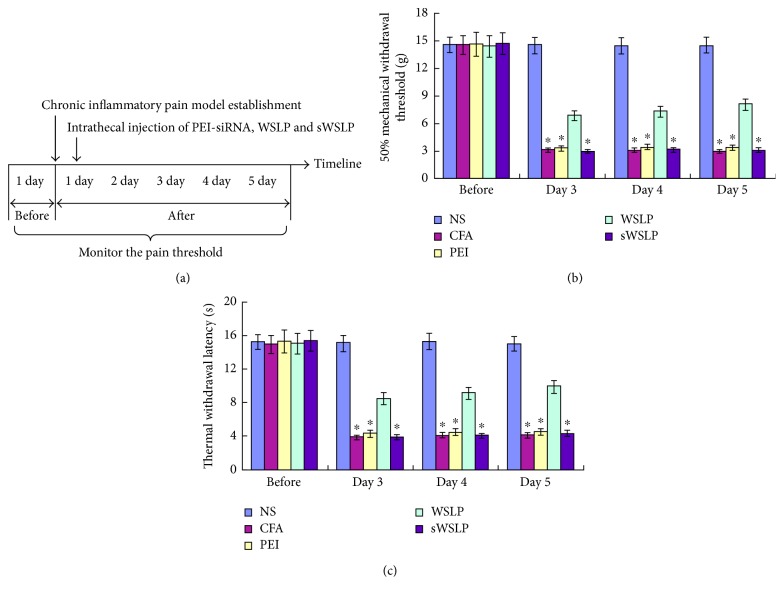
Effects of WSLP/siRNA intrathecal injection on rats with inflammatory pain. Rats were measured the MWT and TWL 2 days before and 3, 4, and 5 days after intrathecal injection. (a) A detailed timeline of animal study during scoring MWT and TWL. (b) The MWT of rats was significantly reduced after the intraplantar injection of CFA and can be effectively increased by intrathecal injection of WSLP/NR2B-siRNA. (c) The TWL of rats also showed a significant decrease after the intrathecal injection of CFA, and intrathecal injection of WSLP/NR2B-siRNA can effectively increase the TWL compared with that in the CFA group. NS: no treatment received; CFA: intraplantar injection of complete Freund's adjuvant; PEI: intrathecal injection of PEI/NR2B-siRNA; WSLP: intrathecal injection of WSLP/NR2B-siRNA; sWSLP: intrathecal injection of scrambled siRNA; MWT: paw mechanical withdrawal threshold; TWL: paw thermal withdrawal latency. ^∗^*p* < 0.01 versus CFA group.

We assessed the MWT and TWL of the rats, which reflect the degree of inflammatory pain of rats. The detailed timeline of animal model establishment was shown in Figure 4(a). Specifically, significantly increased MWT and TWL indicated less pain. We found that the MWT (Figure 4(b)) and the TWL (Figure 4(c)) were significantly reduced in the affected feet of the CFA group rats compared with the NS group rats at 4 days after model establishment via CFA injection (3 days after intrathecal injection) (*p* < 0.01). Moreover, these changes were maintained for 5 days after the intrathecal injection. The increase in MWT and TWL compared with that in the CFA group was the greatest in the WSLP group (*p* < 0.01). The MWT and TWL values for the PEI and sWSLP groups were similar to those for the CFA group (*p* > 0.05). These findings indicated that intrathecal injection of WSLP/NR2B-siRNA relieved inflammatory pain in our rat model.

## 4. Discussion

The key to successful siRNA transfection in vivo is finding an appropriate vector with nontoxicity and high efficiency. Aigner et al. [24] believed that the most viable way is to directly link siRNA with the right vector. Recent lines of evidence discovered that WSLP has shown promise as a genetic carrier with high efficiency and low toxicity [13]. Moreover, WSLP consisted of polyethylenimine (PEI1800) and cholesterol. PEI1800 was also called low molecular weight polyethylenimine, which had lower cytotoxicity and transfection efficiency, compared with PEI25000 (high-molecular-weight polyethylenimine). Previous studies have proved that the biological characters of PEI with different molecular weights are different [25]. (1) PEI of high molecular weight (∼25,000) exhibited better transfection efficiency than PEI of low molecular weight (<5000) [26–28]; (2) PEI of high molecular weight (∼25,000) had higher toxicity to cells, and PEIs can be hampered by their cellular toxicity in vivo. The results in our study were consistent with this, as the PEI synthesized here (∼1800) exhibited low toxicity to cells but not satisfied the transfection effect in vivo. Through modification, PEI (1800) was conjugated with cholesterol to construct the WSLP, and the efficiency was significantly improved.

Of note, the transfection efficiency significantly increased when PEI1800 conjugated to cholesterol [29]. Consistently, in our study, the result showed that WSLP/siRNA has a high stability in serum. NR2B expressions at mRNA and protein levels in the PEI and WSLP groups were lower than those in the NS and sWSLP groups. The cytotoxicity test results showed that, at mass ratios of WSLP/siRNA 1 : 1–7 : 1, the ODs obtained and observed in the MTS tests were not significantly different between WSLP/NR2B-siRNA-transfected and nontransfected cells. These results suggested that WSLP has a low cytotoxicity to cells and a good stability in serum, which entitled favorable value in application.

As we all know, the ratio of carrier and siRNA played a key role in the siRNA transfection, which can significantly affect the transfection efficacy [30]. Zhang et al. [31] found that when the ratio of G5 PD dendrimer to siRNA increased, the efficacy of transfection was upregulated and the expression of target gene was decreased, and 3.5 : 1 was the optimal ratio for silencing the gene expression. In the present study, we synthesized WSLP based on the method mentioned by Lee for example, [16] and discovered that this polymer can effectively bind NR2B-siRNA when they were mixed at a mass ratio of 5 : 1. We then tested the stability of WSLP/siRNA complexes in serum and found that WSLP/NR2B-siRNA still produced a visible electrophoresis band after incubation in serum for 3 h, indicating that it was not degraded.

Previous reports have proved that inhibiting NR2B gene expression contributed to chronic inflammatory pain relief. In this study, we investigated whether WSLP could deliver NR2B-siRNA to successfully inhibit NR2B gene expression in cells and consequently reduce chronic inflammatory pain in the rat model. We found that WSLP/NR2B-siRNA complexes reduced the NR2B transcriptional level and the protein level compared with unmodified siRNA in PC12 cells. There were no significant differences between the NR2B mRNA or protein expression levels of the sWSLP and untransfected groups. These results indicated that WSLP could deliver siRNA to effectively inhibit the genetic expression of NR2B in PC12 cells.

Intrathecal injection was an effective route of administration to avoid recognition and elimination of drugs for gene therapy by the reticuloendothelial system in the blood [32, 33]. Therefore, we aimed to observe the influence of WSLP/NR2B-siRNA on NR2B gene expression and its therapeutic effect on chronic inflammatory pain. The results showed that NR2B protein expression was efficiently inhibited by intrathecal injection of WSLP/NR2B-siRNA complexes; the protein level was reduced compared with that in untreated rats with chronic inflammatory pain. In contrast, injection of WSLP complexed with control scRNA or of PEI with NR2B-siRNA did not produce this inhibitory effect, indicating that the intrathecal injection of WSLP/NR2B-siRNA complexes can specifically inhibit NR2B gene expression in rats with chronic inflammatory pain. Thus, WSLP can efficiently deliver siRNA targeting NR2B in vivo to inhibit NR2B gene expression in the spinal dorsal horn. In addition, the MWT and TWL scores were significantly decreased at each time point in the CFA-injected paw compared with those in normal rat paws. Intrathecal injection of WSLP/NR2B-siRNA complexes lessened the MWT and TWL reduction caused by CFA injection, while the intrathecal injection of PEI/siRNA and WSLP/scRNA did not exhibit such an effect. Thus, the intrathecal injection of WSLP/NR2B-siRNA complexes relieved chronic inflammatory pain in rats.

## 5. Conclusion

It is the first study to confirm that WSLP can efficiently deliver siRNA targeting NR2B to inhibit NR2B gene expression in the spinal dorsal horn and relieve chronic inflammatory pain in rats. It has been reported that intrathecal injection of NR2B-siRNA can relieve nociception of chronic inflammatory pain in rats but is unable to relieve the hyperalgesia of rats with chronic neuropathic pain. siRNA targeting the gene subunit is easily degraded by enzymes and is unable to enter cells, which restricts the therapeutic effect [32, 34]. Wu treated chronic inflammatory pain in rats using adenovirus vector-mediated siRNA to silence NRs [35]. However, the heterologous defect of viral vectors remains an unsolved concern. For example, Garraway et al. [36] found that heterologous HIV-siv vector had a defect in the transduction of dendritic cells (DCs) and macrophages. Although further studies on WSLP/NR2B-siRNA are needed to confirm its use as a nontoxic and high-efficiency vector, our study provides a novel approach to gene therapy, targeting NRs for the treatment of chronic inflammatory pain.

## Figures and Tables

**Table 1:** Expression of NR2B mRNA and protein levels in transfection groups.

	Negative transfection group^a^	Control transfection group^b^	WSLP transfection group^c^
mRNA level^d^	0.69 ± 0.18	0.64 ± 0.13	0.35 ± 0.21^∗^
Protein level^e^	4.36 ± 1.02	4.32 ± 1.09	1.96 ± 0.48^∗^

^∗^ *p* < 0.01 versus the negative transfection group; ^a^PC12 cells transfected with NR2B-siRNA; ^b^PC12 cells transfected with WSLP/scRNA; ^c^PC12 cells transfected with WSLP/NR2B-siRNA; ^d^the mRNA level was calculated by the ratio of NR2B mRNA to *β*-actin mRNA; ^e^the protein level was calculated as the product of the absorbance and area (OD × mm^2^). All values represent the mean ± standard deviation.
